# Anasarca

**Published:** 1887-04

**Authors:** Rush Shippen Huidekoper

**Affiliations:** Dean Veterinary Department, University of Pennsylvania


### ART. X.—ANASARCA.

BY RUSH SHIPPEN HUIDEKOPER.

*Dean Veterinary Department, University of Pennsylvania.*

*SYNOMYMS: Purpura Hæmorrhagica, Scarlatina, Diarrhæmia (Williams), Diastaéshæmia (Delafond), Fiévre Petechiale, Blutfleckenkrankheit, Mal de tete de contagion, Morbus Macalosus.*

BIBLIOGRAPHY. TRASBOT, WILLIAMS, DIECKERHOFF, ROBERTSON, BRUCKMULLER, ETC.

### DEFINITION.

Anasarca, derived from two Greek words—ἀνά, around, and σαρξ, flesh—is strictly an œdema or dropsy of the tissues, an infiltration of serum into the spaces of the connective tissue. It occurs in two forms, essential and symptomatic; the latter is not due to any primary lesion of the tissue affected, but is produced by obstruction in some distant part, to the current of blood, as in some of the heart diseases, tumors, pregnancy, etc., which mechanically increases the pressure in the veins and causes the serous exudation in the part. This form is purely local and will get well spontaneously if the cause can be removed.

Essential Anasarca is a much more serious trouble. It gives rise to lesions so varied in appearance, while identical in their anatomical alterations, it is rendered grave by such extensive and fatal complications and in it, the constitutional phenomena are so unequal in different cases, that the pathology has been variously interpreted and frequently misunderstood. Some authors from certain of the predisposing causes and from the frequent complications of this disease by septicæmia have placed it among the putrid fevers and have considered that it is, at least an infectious, if not a contagious disease. Others, from the appearance of some of the lesions, have made it an eruptive fever and class it as a contagious trouble.

Essential Anasarca, or simply Anasarca, as this term indicates the most frequent lesion without prejudicing in any way, as to the causes of this disease, may be defined as a vaso-motor paralysis, attended by exudation of serum in the connective tissue spaces, either subcutaneous, interstitial or sub-mucous; the exudation frequently accompanied by rupture of the capillaries and extravasation of blood, which at a later period produces constitutional phenomena or fever. The fever is merely the result of the local irritation produced by the lesions and absorption of the exudation.

ETIOLOGY.

Anasarca is confined almost exclusively to the horse and mule, more rare in the latter, but has been seen in the ox and in man. Among the predisposing causes, are cold and wet climates, damp, rainy weather, improperly drained stables, cold and damp from want of ventilation, previous disease of a debilitating character, such as exhaustive pneumonias, pleurisy, severe strangles and influenza. The last disease is the indirect cause, as frequently as all the others combined. The predisposing debility may be the result of bad hygienic conditions, starvation, improper food, overwork and exhaustion. Whatever may have been the origin of the debility and anæmia which is at times present, it is seen generally in animals which have been of unusual force before the predisposing trouble weakened their constitutions. It is seen in the large, heavy draught horses of sanguinary temperament, good feeders, plethoric, strong, active horses of medium size, and but rarely in the fine, clean, dry tissues of the thoroughbred. The cause is usually exposure to cold, rain, snow-storms, draft of air on a heated, but not necessarily a sweating animal. (Fedoré, an Italian physician, mentions a case occurring in a grenadier, who plunged into one of the cold lakes of Northern Italy, after a long march.) In resumé, the etiology is found in a weakened vaso-motor system, which when acted upon by an accidental irritant, is overtaxed, then relaxes and is unable to regain its power over the distended capillaries.

SYMPTOMS.

The symptoms are excessively variable. The local lesions may be confined to a small portion of the animal's body and the constitutional phenomena be nul. The appearance and gravity of the local lesions may be so unlike, from difference of location, that they seem to belong to a separate disease, and complications may completely mask the original trouble.

### PERIOD OF INVASION.

In the simplest form the first symptom noticed is a swelling or several swellings occurring on the surface of the body, on the fore-arm, the leg, the under surface of the belly, or on the side of the head. The tumefaction is at first the size of a hen's egg; not hot, little sensitive and distinctly circumscribed by a marked line from the surrounding healthy tissue. These tumors gradually extend until they coalesce, and in a few hours have swelled up the legs, legs and belly, or the head to an enormous size; they have always the characteristic constricted border, which looks as if it had been tied by a cord. In the nostrils are found small reddish spots or petechiæ, which gradually assume a brownish and frequently a black color. Examination of the mouth will frequently reveal similar lesions on the surface of the tongue, along the lingual gutter and on the frænum. If the external swelling has been on the head, the petechiæ of the mucous membrane are apt to be more numerous and to coalesce into patches of larger size, than when the dropsy is confined to the legs. The animals may be rendered stiff by the swelling of the leg, or be annoyed by the awkward swollen head, which at times may be so enormous as to resemble that of a hippopotamus, rather than that of a horse. During this period, the temperature rem ins normal, the pulse, if altered at all, is only a little weaker, the respiration is only hurried if the swelling of the head infringes on the calibre of the nostrils. The appetite remains normal. The animal is attentive to all that is going on and, except for the swelling, apparently in perfect health.

### STADIUM.

In from two to four days, the tissues can no longer resist the pressure of the exuded fluid. Over the surface of the skin which covers the dropsy, we find a slight serous sweating, which loosens the epidermis and dries, so as to simulate the eruption of some cutaneous disease. If this is excessive, we may see irritated spots which are suppurating. In the nasal fossæ, the hæmorrhagic spots have acted as irritants, and inviting an increased amount of blood to the Schneiderian membrane, produce a coryza or even a catarrh. We may now find some enlargement and peripheral œdema of the lymphatic glands, which are fed from the affected part. The thermometer indicates a slight rise in the body temperature, while the pulse and respiration are somewhat accelerated. The appetite usually remains good. In the course of a few days the temperature may have reached 39° C. or 39.5°, or 40° C. (104° Fahrenheit.)

Fever is established, not an essential or specific fever in any way, but a simple secondary fever produced by the dead material from the surface or superficial suppuration, and by the oxydization and absorption of the colloid mass contained in the tissue. Just such a fever as would be produced by an excoriation of a considerable surface of the skin in an animal, otherwise sound, or by the absorption of the œdema resulting from a blow.

### COMPLICATIONS.

*Suppuration* may become excessive from the great distension and loss of vitality of the skin. Here the lesion is visible and the constitutional phenomena are marked.

*Lymphangitis* may be established from the large amount of irritating material which the ducts and glands of the lymphatic system are forced to carry from the affected part.

*Gangrene* may be developed in spots from the size of a pea to that of a hen's egg. The great distension of the subcutaneous layer of connective tissue or the excessive hæmorrhage in the submucous layer may completely destroy the vitality of the part, and brown and then black masses of a slough appear, to eliminated, and leave a deep rosy ulcer in their place. This is more common in the nose and under the tongue.

*Excessive Swelling of the Head.*—The swelling of the head may increase and extend outside to the throat, or to the nostrils, until the latter are closed, or to the larynx, which is so pressed upon as to render respiration difficult or impossible. The same complication renders mastication and deglutition equally difficult.

*Metastasis.*—This is a frequently dreaded complication. If the trouble has originally been in the legs and belly, it may suddenly commence to appear in the head, and disappear from the part first affected, or the opposite more favorable change sometimes occurs, the dangerous swelling of the head disappearing to attack the belly or legs.

*Enteric Œdema.*—The effusion on the exterior may take another course and pass to the intestine, causing symptoms of colic, which either ends fatally or more rarely terminates by a profuse diarrhœa, which is sometimes hæmorrhagic in character.

*Pulmonary Œdema.*—Marked dyspnœa without swelling of the head, is indicative of metastasis into the parenchyma of the lungs, which will rapidly show dullness on percussion and tubular murmurs on auscultation. The tumefaction leaves the exterior and attacks the lungs and the animal dies of asphyxia.

*Septicæmia.*—There is certainly no disease in veterinary practice which offers a more favorable field for the development of septicæmia. The large mass of colloid matter held at the temperature of the animal body, could not be surpassed in the gelatine tube of a bacteriological laboratory, as a nutriment for the putrefactive ferments. Septicæmia is ushered in by general rigor, sudden elevation of temperature and marked symptoms of coma.

### TERMINATIONS.

*Resolution.*—The simple form of the disease most frequently terminates favorably on the eighth to tenth day by absorption of the effusion, with usually a profuse diuresis and with or without diarrhœa. The appetite remains good or is at times capricious. The surface of the body is dirty from desquamated epithelium, and at times there is complete loss of hair, giving the appearance of a bad case of sarcoptic mange. At other times the absorption is slow, lasting for some weeks with tendency to relapses. Again there may be left some permanent induration the result of embryonic growth.

*Death.*—Death may occur from *mechanical asphyxia*, produced by closure of the nostrils or closure of the glottis. Metastasis to the lungs is almost invariably fatal, causing death by asphyxia. Metastasis to the intestines may cause death from pain, enteritis or hæmorrhage.

*Excessive Suppuration*, *Lymphangitis*, and *Gangrene* are causes of a fatal termination by exhaustion. Mortal exhaustion is again produced by inability to swallow in cases of excessive swelling of the head.

*Peritonitis* may arise secondary to the enteric œdema or by perforation of the stomach or intestines by a gangrenous spot.

*Septicæmia* terminates fatally with its usual train of symptoms.

ANATOMICAL ALTERATIONS.

The essential alterations of anasarca are exceedingly simple; the capillaries are dilated, the lymphatic spaces between the fibres of the connective tissue are filled with serum, and the coagulable portion of the blood, presents a yellowish or citrine mass, jelly-like in consistency, which has spread out the tissue like the meshes of a sponge. Where the effusion has occurred between the muscles, as in the head, these are found dissected and separated from each other, like those of a hog's head by the masses of fat. The surface of the skin is desquamated and frequently denuded of the hair; frequently there are traces of suppuration and of ulceration. The mucous membrane of the nose is found studded with small hæmorrhagic spots, sometimes red, more frequently brown or black, often coalesced with each other in irregular sized patches and surrounded by a reddish zone, the product of irritation. If œdema of the intestines has occurred, the membrane is found four or five times its normal thickness, reddish in color, with hæmorrhages on the free surface. Œdema of the lungs leaves these organs distended. On section a yellowish fluid runs out, like from lungs which have been filled with water in the dissecting room. The secondary alterations vary according to the complications. There are frequently the lesions of asphyxia, externally we find ulcers, abscesses and gangrenous spots and the deep ulcers resulting from the latter. The lymphatic cords and glands are found with all the lesions of lymphangitis. Again are found the traces of excessive emaciation, or the lesions of septicæmia. Except from the complications the blood is not altered in anasarca. If previous to the attack the animal had ænemia, the tissues will be infiltrated and the pallor and other appearances of ænemia will be found. If prior to the attack the animal is in moderate health, with unaltered blood, the blood will be found to clot with the typical change of the buffy coat of the horse. In death by asphyxia the blood will be found fluid, black in color but gradually turn red, and clot on exposure to the air. Dickerhoff mentions fibrinous pneumonia among the alterations. I myself have never seen it occur.

### DIAGNOSIS

must principally be made from farcy-glanders. In anasarca the swelling is non-sensitive, while sensitive in the acute swelling of farcy. The nodes of farcy are distinct and hard and never circumscribed as in the other disease. The eruption of glanders on the mucous membrane is nodular, hard and pellet-like. The redness disappears on pressure. The petechiæ of anasarca are not elevated, are due to extravasated blood and do not change color on pressure. In cases of excessive swelling of the head in anasarca there may occur an extensive sero-fibrinous exudation from the mucous membrane of the nose, poured out as a semi-fluid mass or as a cast of the nasal fossæ, never having the appearance or typical oily character which it has in glanders. The inflammation of the lymphatic cords and glands in anasarca does not produce the hard indurated character, which is found in farcy.

*Septicæmia* may have occurred primarily or as a complication of anasarca. The diagnosis must be made from the history, and the prognosis is of little import.

### PROGNOSIS.

While this is not an excessively fatal disease the prognosis must always be guarded. The majority of cases run a simple course and terminate favorably at the end of eight or ten days or possibly after one or two relapses, requiring several weeks for complete recovery. Effusion into the head renders the prognosis much more grave, from the possible danger of mechanical asphyxia. Threatened mechanical asphyxia is especially dangerous on account of the risk of blood-poisoning after an operation of tracheotomy.

Metastasis to the viscera or from the legs to the head is a most serious complication, while metastasis from the head or other portions to the belly and legs, is favorable, as removing, for the moment at least, all the danger of immediate death. The prognosis is otherwise based on the complications, their extent and their individual gravity, existing, as they do here, in an already debilitated subject.

TREATMENT.

The treatment of anasarca may have to be as variable, as are the lesions. The indications are at once shown by the alterations, and mechanism of the disease, which we have just studied. They are:

1st. Regulation of the disordered circulation of the blood and strengthening of the vaso-motor system.

2d. Promotion of absorption of the colloid mass, which has infiltrated the tissues. This of course is based upon oxydization in order to metamorphose the exudation into absorbable crystalloids.

3d. Prevention of metastasis, which is the most frequent fatal termination of this trouble; if not directly by œdema of the lung or enteritis, indirectly by further weakening the already debilitated system.

4th. The immediate treatment of the complications, each *per se*; asphyxia and gangrene being the two which most frequently call for active interference.

These indications call for constitutional and local remedies.

CONSTITUTION TREATMENT.

*Blood letting* would at first seem totally contra-indicated, but in certain cases it acts like a charm. Debilitated as the animal usually is when attacked by anasarca, we have yet seen that one of the great predisposing causes is the plethoric habit. The current of blood, like a swollen river after a spring storm, can be thrown from its usual course by the slightest side channel. The use of bleeding requires the acute perception of the practitioner to be put upon the alert to regulate it. Not only the present condition, but the previous state of health, and the probable future hygienic and medical care must be taken into consideration. Given a case that will admit of bleeding, the quantity to be taken is always a minimum one, and it is to be regulated by the quality of blood that is found.

With the weakened walls of the vessels but a little lessening of the pressure will produce a vacuum, when compared with the condition found in an ordinary blood vessel system, with normal elastic walls. Bleeding is only permissable at the outset of the disease, when the tumors are still isolated. When the tumefaction has coalesced, all the blood is required to oxydize the mass of effused colloid matter.

*Hygiene* now comes into play as the most important factor. Oats, oat and hay tea, milk, eggs, anything which the stomach or rectum can be coaxed to take care of, must be employed to to give the nutriment which is the only thing which will permanently strengthen the tissues, and they must be strengthened in order to keep the capillaries to a proper calibre.

Laxatives, diaphoretics and diuretics must be used to stimulate the emunctories so that they shall carry off the large amount of the products of decomposition, which result from the stagnated effusions of anasarca. Of these the sulphate of soda in small repeated doses, the nitrate of potash and bicarbonate of soda in small quantity, and the chlorate of potash in single large doses, will be found useful. Williams cites the chlorate of potash as an anti-putrid, it is useful but I believe because it frees oxygen—and oxygen is a chemical purifier.

Stimulants and astringents are directly indicated. The animal wants wakening up, everything in it wants a shock, and a belt to hold it in place. Spirits of turpentine serves the double purpose of a cardiac stimulant and a powerful warm diuretic; for the kidneys in this disease will stand a wonderful amount of work. Camphor can be used with advantage. Coffee and tea are two of the diffusible stimulants which are too much neglected in veterinary medicine; both are valuable adjuncts in treatment in anasarca, as they are during convalescence, at the end of any grave disease, which has tended to render the patient anæmic. Dilute sulphuric acid and hydrochloric acid are perhaps the best examples of a combination of stimulant, astringent and tonic which can be employed. The simple astringents of mineral origin, sulphates of iron, copper, etc., are useful as digestive tonics ; I doubt if they have any constitutional effect. The vegetable astringents, tannic acid, etc., have not proved efficacious in my hands. Iodide of potash in small doses serves the triple purpose of digestive tonic, denutritive for inflammation and diuretic.

*Externally*—sponging the swollen parts, especially the head, when the swelling occurs here, is most useful. The bath should be at an extreme of temperature—either ice water, to constrict the tissues, or hot water, to act as an emollient and to favor circulation. Vinegar may be added as an astringent. When we have excessively denuded surfaces, suppuration or open wounds, disinfectants should be added to the wash.

In cases of excessive swelling, especially of the head, mechanical relief may be required. Punctures of the part should be made with the hot iron even in country practice, as no other disease so predisposes to septic contamination. When mechanical asphyxia is threatened, tracheotomy may be demanded. Here, again, the hot iron should be used, and disinfectant applications should be constantly applied. With the first evidence of dyspnœa, not due to closing of the nostrils or glottis, or with the first pawing which gives rise to a suspicion of colic, a mustard plaster should be applied over the whole belly and thorax. The sinapism will draw the current of the circulation to the exterior, the metastasis to the lungs or intestines is prevented, and the enfeebled, nervous system is stimulated to renewed vigor by the peripheral irritation ; the organs are encouraged by it to renewed functional activity ; the local inflammation produced by it favors absorption of the exdation. The objection to the use of blisters is their more severe action and the danger of mortification. Septicæmia, when occurring as a complication, requires the ordinary treatment for the putrid diseases, with little hope of a good result.

In termination, anamese is misunderstood; etiology is debility; symptoms are mechanical effusions; sequelæ are inflammatory products of irritation; pathology is the result of symptoms and sequelæ; treatment is indicated by etiology, *i. e.*, tonics and stimulants.

